# Detection and Molecular Characterization of Rift Valley Fever Virus in Apparently Healthy Cattle in Uganda

**DOI:** 10.3390/pathogens14070720

**Published:** 2025-07-20

**Authors:** Eugene Arinaitwe, Kaitlyn Waters, Bonto Faburay, Gladys K. Nakanjako, David Kalenzi Atuhaire, Mathias Afayoa, Frank Norbert Mwiine, Joseph Erume

**Affiliations:** 1 National Animal Disease Diagnostics and Epidemiology Centre (NADDEC), Entebbe P.O. Box 513, Uganda; arieugene@yahoo.com (E.A.); gladyskiggundu@gmail.com (G.K.N.); 2 Foreign Animal Disease Diagnostic Laboratory, National Veterinary Services Laboratories, Animal and Plant Health Inspection Service, United States Department of Agriculture, Plum Island Animal Disease Center, Orient, NY 11957, USA; kaitlyn.waters24@gmail.com; 3 Foreign Animal Disease Diagnostic Laboratory, National Veterinary Services Laboratories, Animal and Plant Health Inspection Service, United States Department of Agriculture, National Bio and Agro-Defense Facility, Manhattan, KS 66502, USA; 4 College of Veterinary Medicine, Animal Resources and Biosecurity, (COVAB), Makerere University, Kampala P.O. Box 7062, Uganda; kalenzid@gmail.com (D.K.A.); mafayoa@gmail.com (M.A.); fmwiine@gmail.com (F.N.M.); joseph.erume@mak.ac.ug (J.E.)

**Keywords:** Rift Valley fever, domestic ruminants, genome sequencing, genetic diversity, zoonotic, phylogenetic analysis

## Abstract

Rift Valley fever (RVF) is a zoonotic disease caused by the Rift Valley fever virus (RVFV), affecting humans, livestock, and wild ruminants. This study aimed to characterize and assess the genetic diversity of RVFV strains circulating among livestock in Uganda. Blood samples were collected between January 2021 and May 2024 from apparently healthy cattle, goats, and sheep in four districts. The samples were first screened for RVFV antibodies using ELISA; antibody-positive samples were subsequently tested for viral RNA using reverse transcriptase quantitative PCR (RT-qPCR). The PCR-positive samples underwent targeted amplicon sequencing, and phylogenetic analyses of the small (S) and large (L) genome segments were conducted to determine viral lineages. Of the 833 ELISA-positive samples, 10 (all from cattle) tested positive for RVFV RNA using RT-qPCR. Consensus sequences were successfully generated for six S segments and one L genome segment. A phylogenetic analysis revealed that all sequences belonged to lineage C, showing close genetic similarity to RVFV strains previously identified in Uganda, Kenya, Sudan, Madagascar, and Saudi Arabia. Limited genetic diversity was observed at both the nucleotide and amino acid levels. The detection of RVFV in apparently healthy cattle suggests ongoing, low-level viral circulation in Uganda. These findings offer important insights for guiding RVF surveillance, control, and policymaking in the country.

## 1. Introduction

Rift Valley fever (RVF) is an emerging viral, vector-borne zoonotic disease transmitted primarily by mosquitoes that affects humans as well as domestic and wild ruminants, such as cattle, sheep, goats, and buffalo [1]. Humans become infected when bitten by infected mosquitoes or through contact with the bodily fluids of an infected animal. The disease is caused by the Rift Valley fever virus (RVFV), which belongs to the family *Phenuiviridae* of the order *Bunyavirales* and genus *Phlebovirus* [2,3]. RVFV was first isolated in 1930 from a dead sheep in Kenya [4]. The disease occurs in many African countries; by the year 2000, RVFV had spread beyond Africa, causing a major outbreak in Saudi Arabia and Yemen [5]. Outbreaks of RVF often occur following periodic heavy rains and sustained flooding. To date, cases of RVF are known to occur on the continents of Africa and Asia. RVF was first detected in Uganda in 1944, when it was identified in mosquitoes collected from the Semliki Forest in the western part of the country [6]. Later, in 1955, the Lunyo strain was isolated from a pool of *Aedes* mosquitoes in the Lunyo Forest in Entebbe [7]. Uganda reported its first RVF outbreak in 2016 in the Kabale district, and since then the disease has spread to several other districts of the country [8].

RVF is often characterized by high rates of abortion and neonatal mortality, especially in livestock [9]. The Rift Valley fever virus genome consists of three negative-sense, single-stranded RNA segments categorized as L (large), M (medium), and S (small). The S segment has a size of 1690 nucleotides (nt), the M segment has 3885 nt, and the L segment has 6404 nt. The total combined genome size is approximately 11.9 kb [10,11]. The L segment encodes the RNA-dependent RNA polymerase, and the M segment encodes the precursor protein of two structural glycoproteins, Gn and Gc, which are present on the virus surface. Cleavage of the precursor protein leads to two additional non-structural proteins of 78 kDa (P78 or LGp) and 14 kDa (NSm) in molecular mass. Studies have shown that the Gn and Gc are involved in the attachment of the virus to the host cell, and the NSm is known to inhibit apoptosis but is not essential for RVFV replication and virulence [12,13]. The LGp/P78 protein is a major determinant of virus dissemination in mosquitoes [14]. The S segment encodes the structural viral nucleocapsid protein, or N protein, and nonstructural NSs protein [11]. The N protein encapsulates viral RNA in a nucleocapsid which is necessary for transcription and RNA replication, and the NSs protein has been shown to inhibit host innate immune responses [15].

Rift Valley fever virus is known to consist of one serotype; however, several strains do exist with variable virulence. Next-generation sequencing has made it possible to determine and better understand the genetic diversity of RVFV, which is necessary to develop reliable diagnostic methods, support the development of antivirals and vaccines, and identify the source of new disease outbreaks. This can be achieved by performing whole genome or targeted amplicon sequencing and phylogenetic analysis to enhance the understanding of the continuing evolution of RVFV [11]. To date, the number of identified viral lineages is 15 (A to O) [16]. The Lunyo strain (accession numbers KU167025-KU167027) clusters with other strains originating from Uganda within lineage E. These strains are closely related to the Entebbe strain (accession numbers DQ380156, DQ380191, and DQ375429) isolated from mosquitoes in Entebbe, Uganda, in 1955 [7]. Other RVF viruses belonging to lineages K and M were isolated in 1944 and 1955, respectively, from Uganda [11].

The continuous spread of RVFV in several districts of Uganda, since the virus’ reemergence in 2016, is of great concern [8]. Therefore, the purpose of this study was to conduct a molecular epidemiological investigation among livestock in Uganda to aid the understanding of potentially circulating RVFV strains and virus evolution in the country to inform control measures.

## 2. Materials and Methods

### 2.1. Study Area

This study was conducted in the districts of Isingiro, Kiruhura, Nakasongola, and Gomba, which are all located along Uganda’s cattle corridor. The GPS coordinates for each sample collection site are detailed in Supplementary Table S1. This corridor is a broad zone that stretches from the southwestern to the northeastern region of Uganda and is noted for its agricultural and pastoral activities.

### 2.2. Sample Collection and Processing

Whole blood samples were collected in EDTA tubes from apparently healthy cattle, goats, and sheep. The samples were labeled with a field identification code. To obtain the serum, blood samples were allowed to clot, and the serum was separated. The samples were preserved on ice in a cool box and transported to the National Animal Disease Diagnostics and Epidemiology Centre (NADDEC), Entebbe, where they were stored at −20 °C until analyzed. After analysis, all samples were transferred to the −80 °C freezer for long storage.

A total of 3785 samples (bovine, n = 1963; caprine, n = 1282; ovine, n = 540) were collected between January 2021 and May 2024. These samples were screened for RVFV IgG antibodies by ELISA, using a Rift Valley fever Competitive Multi-species ID Screen IDVet ELISA kit. All the IgG-positive samples were tested for IgM antibodies using the Rift Valley fever IgM Capture kit (ID Screen, IDVet-Innovative Diagnostics, 310, Rue Louis Pasteur, 34790 Grabels, France). Of the total samples screened, 833 tested positive for RVFV IgG and 32 for IgM antibodies. To verify the presence of the RVF virus, all 833 samples that showed IgG positive results in the ELISA test were subsequently analyzed using RT-qPCR as outlined below.

### 2.3. RNA Extraction

RNA was extracted from saliva and scrapings of tongue epithelial tissue and the residual serum or whole blood (due to limited serum availability after conducting ELISA and several PCR assays) of cattle, goats, and sheep. The extraction was performed using a QIAamp Viral RNA Mini kit (QIAGEN, Valencia, CA, USA) following the manufacturer’s instructions. Briefly, 140 μL of the sample was added to 560 μL AVL buffer containing carrier RNA into a 1.5 mL micro-centrifuge tube and mixed by pulse-vortexing for 15 s followed by incubation at room temperature for 10 min. Protein precipitation was accomplished by adding 560 μL of absolute ethanol and mixing by pulse-vortex for 15 s. The lysate was then passed through a mini spin column, and the column was washed twice with 500 μL of washing buffers AW1 and AW2. RNA was eluted by adding 60 μL of buffer AVE. The extracted RNA was stored in a freezer at −80 °C until required for further analysis.

### 2.4. Reverse Transcriptase Quantitative PCR (RT-qPCR)

The extracted RNA was reverse transcribed and amplified using a SuperScript III One-Step RT-PCR with Platinum Taq kit (Invitrogen, Carlsbad, CA, USA). The following primers and probes were used to amplify 90 bp of the L segment of the viral genome: forward primer—RVF-2912fwd, 5′-TGA AAA TTC CTG AGA CAC ATG G-3′; reverse primer—RVF-2981rev, 5′-ACT TCC TTG CAT CAT CTG ATG-3′; and probe—RVFL-probe-2950; FAM-5′-CAA TGT AAG GGG CCT GTG TGG ACT TGT G-3′-BHQ1 [6]. Samples were run on an ABI 7500 FAST, Real-Time PCR machine using the following conditions: 50 °C for 10 min, 95 °C for 2 min, and 45 cycles of 95 °C for 10 s and 60 °C for 60 s. The purified RNA from the 10 RT-qPCR-positive samples were shipped to the Foreign Animal Disease Diagnostic Laboratory (FADDL) at Plum Island Animal Disease Center, USA, for next-generation sequencing.

### 2.5. Next-Generation Sequencing

The extracted RNA was reverse transcriptase (RT)-PCR amplified using the SuperScript^TM^ III One-Step RT-PCR System with Platinum^TM^ *Taq* High Fidelity DNA polymerase (Invitrogen, San Diego, CA, USA) to generate four individual amplicons for the three RVFV segments as previously described [10]. Briefly, the S and M segments were each amplified as one amplicon, whereas the L segment was amplified into two overlapping amplicons. The purification of individual segment amplicons and pooled segment amplicons was performed using AMPure XP DNA beads (Beckman Coulter, Brea, CA, USA) on the KingFisher FLEX magnetic particle processor with a 96 deep well head (ThermoFisher Scientific, Waltham, MA, USA). Libraries were generated by diluting the purified amplicons to 0.2 ng/µL and using a Nextera XT DNA library Preparation Kit (Illumina, San Diego, CA, USA) and Nextera XT Ind.ex Kit v2 Set A (Illumina, San Diego, CA, USA). Dual-indexed libraries were diluted to 2 nM and pooled for paired-end sequencing on the Illumina MiSeq platform using the 500 cycle v2 reagent kit and flow cell (Illumina, San Diego, CA, USA).

### 2.6. Sequence Data and Phylogenetic Analyses

The sequencing data were analyzed using CLC Genomics Workbench version 24.0 (Qiagen, Carlsbad, CA, USA). Paired FASTQ read files were imported into the CLC software and parsed into individual sample files. Trimmed reads were mapped to the respective reference sequences (S Segment: NC_014395.1; M Segment: NC_014396.1; L Segment: NC_014397.1) using the CLC Genomics Workbench Map Reads to Reference module. The alignment of the obtained consensus S and L segments was performed using the CLC Create Alignment module. Consensus S and L sequences were aligned to other S and L segment sequences downloaded from the NCBI nucleotide database. A diverse group of RVFV strains (n = 49) collected throughout Africa and the Arabian Peninsula spanning 77 years (from 1944 to 2021) were downloaded from NCBI to be included in this analysis. These strains represented the 15 RVFV lineages (A to O) previously identified [16] from a variety of host species, such as mosquitoes, cattle, sheep, bats, and humans. Phylogenetic analyses using maximum likelihood (ML) methods with 1000 bootstrap replicates were conducted.

## 3. Results

### 3.1. Serological and Molecular Detection of RVFV in Livestock

A total of 3785 animals (cattle, goats, and sheep) from four districts in Uganda were tested for Rift Valley fever virus (RVFV) exposure and infection. RVF-specific IgG antibodies, indicating past exposure, were detected in 833 animals (22.0%). Cattle had the highest IgG seroprevalence (31.7%), especially in Nakasongola (43.8%) and Gomba (33.8%). IgM antibodies, which indicate recent infection, were found in 32 animals (3.8%). The highest IgM prevalence was recorded in goats from Isingiro (14.1%) and cattle from Kiruhura (6.5%) and Nakasongola (3.1%) (Table 1).

**Figure 1:**
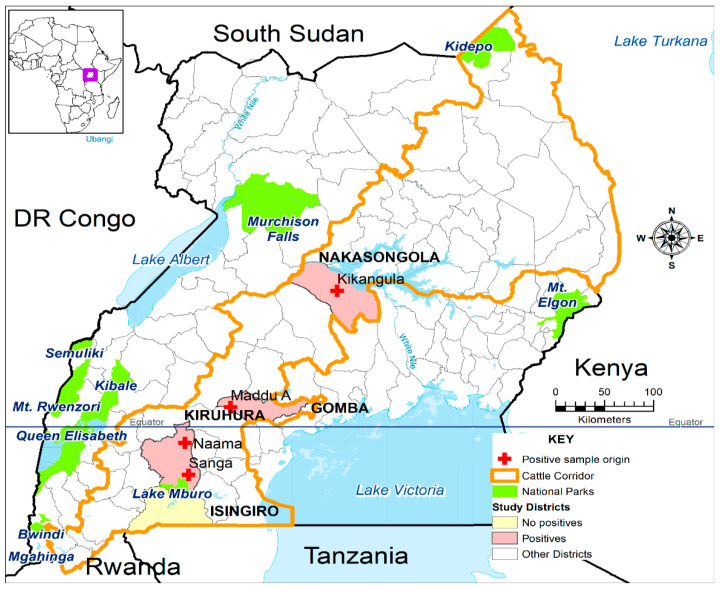
Map of Uganda showing areas where cattle samples tested positive for Rift Valley fever virus with RT-qPCR (Sanga n = 4, Naama n = 1, Maddu n = 2, and Kikangula n = 3). Uganda, located in East Africa is highlighted in purple on the Map of Africa (Top left corner).

Of the 833 animals tested using RT-qPCR, 10 cattle (1.2%) tested positive for RVFV RNA, confirming active infection. All RT-PCR-positive cases were from the Gomba, Kiruhura, and Nakasongola districts. No goats or sheep tested positive using PCR, despite some showing IgM positivity (Table 2). Figure 1 shows the districts in Uganda where positive samples were collected.

### 3.2. Next-Generation Targeted Amplicon Sequencing of RVFV Samples

Here we report the successful next-generation sequencing of RVFV genome segments using a targeted amplicon sequencing method. Of the ten RT-qPCR-positive RVFV samples collected from cattle in Uganda, the S segment was successfully sequenced for six samples and the L segment was sequenced for one sample (Table 3). For the S segment, the targeted amplicon method increased the quality metrics of sequencing output by producing an average depth of coverage ranging from 33,254.7× to 80,925.2×. The total number of mapped reads for the segment ranged from 312,561 to 883,631 with the percentage of reads mapped to the reference segment (percent coverage of the genome segment) ranging from 95.54% to 99.29% (Table 3). These metrics represent ~100% S segment coverage. The average depth of coverage for the L segment was 17,999.4×. The total number of mapped reads was 1,359,265 with the percentage of reads mapped to the reference segment at 70.15% (Table 3). These metrics represent ~100% L segment coverage. To summarize, the one-step RT-PCR amplification of target amplicons resulted in six consensus S segment sequences and one consensus L segment sequence.

### 3.3. Phylogenetic Analysis of the S and L Segments of RVFV Samples from Cattle in Uganda

**Figure 2:**
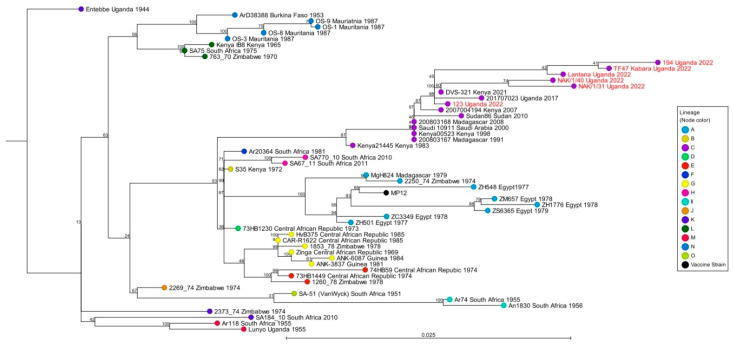
Phylogenetic analyses of RVFV S segment. The RVFV S segment phylogenetic tree was inferred using the CLC Genomics Workbench with 1000 bootstrap replicates. Samples from this study are highlighted in red font.

**Figure 3:**
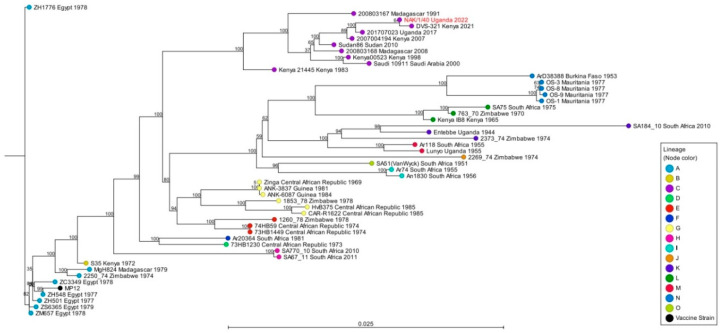
Phylogenetic analyses of RVFV L segment. The RVFV L segment phylogenetic tree was inferred using the CLC Genomics Workbench with 1000 bootstrap replicates. The sample from this study is highlighted in red font.

Phylogenetic analyses of the S segments indicate the six sample cluster in lineage C (Figure 2 and Table 4). Five of these samples (Lantana, TF47 Kabara, 194, NAK/1/31, and NAK/1/40) exhibited 99.70% identity to the DVS-321-Kenya 2021 (OM744371.1) virus, whereas sample 123 exhibited 99.17% identity with the Sudan 86-2010 (JQ820477.1) isolate. As for the L segment, the NAK/1/40 sample showed 99.75% identity with the DVS-321-Kenya 2021 (OM744376.1) isolate, and clustered in lineage C (Figure 3 and Table 4).

### 3.4. Genetic Variation In S Segments of RVFV Samples from Cattle in Uganda

The S segment contains two open reading frames (ORFs) encoding the NP and NSs proteins. The least amount of variation was observed for the S segment ORF encoding the NP protein as no changes were observed at the nucleotide or amino acid level among the samples. At the nucleotide level of the NSs protein, the maximum pairwise percent identity varied from 0.12% (or 2 nucleotide differences) to 0.97% (or 16 nucleotide differences). These differences at the nucleotide level translated to four amino acid differences and were observed only in sample 123 (Table 5), whereas the NSs proteins of the remaining five samples were identical.

## 4. Discussion

This study provides compelling evidence of both the historical exposure and ongoing transmission of Rift Valley fever virus (RVFV) among domestic ruminants in four ecologically diverse districts of Uganda. The detection of RVFV-specific antibodies, viral RNA, and the successful sequencing of viral genomes from livestock underscores the endemic nature of the virus in these districts and the potential risk for future RVF outbreaks affecting both animal and human health.

Of the 3785 animals sampled, 22.0% were IgG-positive, indicating widespread past exposure. Cattle had the highest IgG prevalence (31.7%), consistent with their role as primary amplifying hosts and increased exposure to mosquito vectors [17,18]. In contrast, goats and sheep showed lower IgG prevalence (13% and 8%, respectively), potentially due to species-specific vector exposure, immunity, or host–pathogen interactions. Spatially, IgG prevalence was highest in Nakasongola (43.8%) and Gomba (33.8%), indicating endemic transmission or previous outbreaks in these districts. Kiruhura (23.7%) and Isingiro (26.0%) had moderate IgG prevalence, reflecting relatively lower but consistent RVFV circulation. In this study, no attempts were made to isolate infectious viruses from positive samples.

Evidence of recent infections was detected in 3.8% of the animals via IgM seropositivity, with a notably high IgM prevalence in goats from Isingiro (14.1%). However, none of the IgM-positive goats or sheep were PCR-positive, likely due to sampling during the post-viremic phase or species-specific differences in viremia duration, with goats known to clear viremia faster than cattle [19,20]. In contrast, cattle from Kiruhura (6.5%) and Nakasongola (3.1%) exhibited concurrent IgM and PCR positivity, confirming active viral replication and reinforcing their role as key sentinels in RVFV transmission [10,17]. The absence of PCR-positive goats and sheep may reflect a combination of factors, including lower sampling numbers, species-specific viral kinetics, or reduced diagnostic sensitivity in these hosts. Notably, PCR-positive cattle were detected across multiple districts, particularly Gomba, Kiruhura, and Nakasongola, establishing these areas as current transmission hotspots. To assess the genetic diversity of circulating RVFV strains, whole genome sequencing was performed on the 10 PCR-positive samples. The S segment was successfully sequenced from six samples, and the L segment from one, all of which were derived from cattle.

Phylogenetic analyses of the S and L segment sequences from these six samples showed that the RVFV strains circulating among cattle in the region belong to lineage C. Remarkably, RVFV isolates from lineage C have been reported to be highly pathogenic, including the historical strains responsible for the outbreak of 1977 to 1978 that resulted in 598 human deaths in Egypt [10,21]. The presence of potentially highly pathogenic strains of RVFV in the study region in Uganda suggests the potential for RVF epidemics and/or epizootics with major consequences for both animal and public health; therefore, the sensitization of communities and regular surveillance of RVFV to monitor virus evolution, genotypically and phenotypically, should be implemented to prevent and mitigate future outbreaks. Additionally, the extent of the co-circulation of wildtype viruses within lineage C and outside of it should be further investigated to assess the risk of reassortment, which could yield new virus strains with altered genotypic and phenotypic characteristics and the potential to cause disease outbreaks. Remarkably, other studies have reported an increased virulence of RVFV in humans due to virus reassortment [22]; reassortment viruses have been isolated in fatal human cases in Mauritania [23].

The phylogenetic analysis based on the S segment indicated that the six samples clustered within lineage C. Our analysis further demonstrated that the six samples clustered with isolates from Uganda (201707023 Uganda 2017), Kenya (DVS-321 Kenya 2021), Sudan (Sudan86 Sudan 2010), and Saudi Arabia (Saudi 10911 Saudi Arabia 2000). The clustering of local Ugandan samples with the previously noted isolates, as well as the existence of multiple viral genetic lineages within a relatively small geographic area, suggest the introduction and dispersal of different RVFV strains across Africa due to wide intra-continental animal movements (transhumance and trade), and the opportunity for virus reassortment to occur in these regions.

Genetic diversity at both the nucleotide and amino acid levels was low among the six samples. These findings are consistent with earlier research. Notably, in one study, RVF virus diversity was reported to be as low as 5% at the nucleotide level and 2% at the amino acid level [10]. Similarly, the low genetic diversity of RVFV was reported during the outbreaks in 1977 in Egypt, 1983 in Mauritania, and 2006–2007 in East Africa [22]. However, despite low genetic diversity, differences in pathogenesis among different RVFV strains in mammals have been observed [10]. This could potentially be attributed to differences in host responses or the specific viral characteristics [24]. Together, these findings suggest that RVFV evolves at a low mutation rate, and the viruses probably originated from a recent ancestor [10].

Notably, all six RVFV-positive samples in this study were detected exclusively in cattle (bovines), while goats (caprines) and sheep (ovines) tested negative with RT-qPCR. This contrasts with the findings from previous outbreaks in Rwanda (2018, 2022) and Kenya (2006–2007), where RVFV lineage C was identified across all three species—cattle, goats, and sheep [25,26]. Even during interepidemic periods in Kenya, RVFV has been detected in all three species, particularly in sheep and goats [27], which are considered more susceptible to infection. The absence of positive cases in goats and sheep in the present study may reflect differences in sampling strategy, timing, or diagnostic sensitivity, rather than true host specificity. However, further research is needed to determine whether the RVFV lineage C identified here exhibits any host restriction or if other ecological or epidemiological factors contributed to this pattern. The detection of RVFV-positive cattle across multiple districts in Uganda (Figure 1) also indicates a wide geographic distribution and the potential risk for future outbreaks in both livestock and humans. These findings underscore the importance of robust surveillance and sensitive diagnostics to accurately characterize RVFV transmission dynamics and host range.

Furthermore, as the samples in this study belong to lineage C, the genomic characterization shows that the branching does not appear to be monophyletic suggesting that other closely related viruses could be circulating in the country without a novel introduction from another country and could facilitate the emergence of new genetic variants with phenotypic implications. In Uganda, extensive genomic surveillance has not previously been conducted to determine the diversity of the RVFV strains circulating in the country. It is noteworthy that none of the samples were closely related to other historic Ugandan RVFV strains, such as the Entebbe Uganda 1944 (lineage K) and Lunyo Uganda 1955 (lineage E), which were isolated from mosquitoes [28]. This suggests that the samples characterized in this study have either evolved independently of these historical strains or represent new strains introduced from elsewhere through livestock trade or transhumance.

Some limitations need to be considered when interpreting the results of this study. Firstly, there was unequal sampling across livestock species, with a higher number of cattle tested than goats or sheep. The lack of PCR-positive goats and sheep may reflect this sampling bias or sample degradation due to poor handling, rather than true species resistance. It could also be due to low or short transient viremia as well as the potential low survivability of small ruminants to RVFV infection; evidently, small ruminants have been shown to be more susceptible to RVFV than cattle [29,30]. Secondly, this study was limited to only four districts, which may not capture the full extent of RVFV transmission or viral diversity across Uganda. Broader geographic sampling and the inclusion of mosquito vectors and wildlife hosts would provide a more comprehensive picture of RVFV ecology.

## 5. Conclusions

The detection and characterization of RVF viruses in livestock using RT-qPCR and genome sequencing indicate that there is active circulation of RVFV in the study region in Uganda. Further genomic studies are required in humans and livestock to aid in understanding the molecular epidemiology of the disease in the country and also provide opportunities for understanding transmission dynamics and predicting future outbreaks. Furthermore, our study shows evidence of the circulation of different strains of the virus within lineage C; the co-circulation of multiple strains within the domestic livestock population presents a risk of genetic reassortment and the emergence of potentially highly virulent strains that could cause serious disease outbreaks. The fact that the positive samples were obtained from apparently healthy livestock suggests that RVFV could be circulating in Uganda and in other endemic countries unnoticed at low levels; therefore, routine active disease surveillance and the characterization of all confirmed cases should be implemented. This study was limited to only four districts in Uganda; therefore, a wider study would be required to accurately determine the prevalence and characteristics of all of the RVFV strains circulating in Uganda. Our findings could inform future RVF disease control policies and strategies in the country.

## Figures and Tables

**Table 1:** Summary of RVFV IgG, IgM, and RT-qPCR results by species and district.

Species	District	IgG Tested	IgG+ (%)	IgM Tested	IgM+ (%)	PCR Tested	PCR+ (%)
Cattle	Gomba	597	202 (33.8%)	202	4 (2.0%)	202	2 (1.0%)
	Isingiro	412	107 (26.1%)	107	1 (0.9%)	107	0 (0.0%)
	Kiruhura	518	123 (23.7%)	123	8 (6.5%)	123	4 (3.3%)
	Nakasongola	436	191 (43.8%)	191	6 (3.1%)	191	4 (2.1%)
	Subtotal	1963	623 (31.7%)	623	19 (3.0%)	623	10 (1.6%)
Goats	Gomba	243	38 (15.6%)	38	0 (0.0%)	38	0 (0.0%)
	Isingiro	436	85 (19.5%)	85	12 (14.1%)	85	0 (0.0%)
	Kiruhura	368	19 (5.2%)	19	1 (5.3%)	19	0 (0.0%)
	Nakasongola	235	25 (10.6%)	25	0 (0.0%)	25	0 (0.0%)
	Subtotal	1282	167 (13.0%)	167	13 (7.8%)	167	0 (0.0%)
Sheep	Gomba	71	1 (1.4%)	1	0 (0.0%)	1	0 (0.0%)
	Isingiro	234	19 (8.1%)	19	0 (0.0%)	19	0 (0.0%)
	Kiruhura	144	13 (9.0%)	13	0 (0.0%)	13	0 (0.0%)
	Nakasongola	91	10 (11.0%)	10	0 (0.0%)	10	0 (0.0%)
	Subtotal	540	43 (8.0%)	43	0 (0.0%)	43	0 (0.0%)
Overall Total		3785	833 (22.0%)	833	32 (3.8%)	833	10 (1.2%)

**Table 2:** Rift Valley fever virus RT-qPCR positive samples collected from cattle.

Field Sample ID	District	Age (yrs.)	Sex	Breed	Sample Type	Ct Value
Ngabo	Kiruhura	>2	F	Friesian Cross	Whole Blood	29.91
Lantana	Kiruhura	>2	F	Friesian Cross	Whole Blood	41.7
TF47 Kabara	Kiruhura	>2	F	Ankole	Saliva/Epithelial tissue	19.48
194	Kiruhura	>2	F	Ankole	Serum	26.75
123	Kiruhura	<1	F	Ankole	Serum	25.9
NAK/1/23	Nakasongola	<1	M	Boran	Whole Blood	35.62
NAK/1/31	Nakasongola	>1	M	Boran	Whole Blood	37.15
NAK/1/40	Nakasongola	>1	M	Boran	Whole Blood	26.55
GMB/003/04C	Gomba	>2	F	Friesian Cross	Serum	40.04
GMB/004/04C	Gomba	>2	F	Friesian Cross	Serum	34.48

**Table 3:** Summary of next-generation sequencing results of the 6 RVFV samples.

Samples	Segment	Total No. of Reads	Total No. of Target Reads Mapped	% of Target Reads Mapped	Average Coverage	GenBank Accession Numbers
Lantana Uganda 2022	S	338,801	325,609	96.11	34,387.1	PV562141
TF47 Uganda 2022	S	327,164	312,561	95.54	33,254.7	PV562142
194 Uganda 2022	S	891,577	883,631	99.11	80,925.2	PV562143
123 Uganda 2022	S	424,999	421,844	99.26	41,029.9	PV562144
NAK/1/31 Uganda 2022	S	488,934	485,470	99.29	49,587.9	PV562145
NAK/1/40 Uganda 2022	S	368,564	365,504	99.17	33,484.9	PV562146
	L	1,937,608	1,359,265	70.15	17,699.4	PV562147

**Table 4:** Genomic characterization of RVFV samples from cattle in Uganda.

Sample	Segment	Nearest Segment (% Identity NT) ^a^	Lineage
Lantana Uganda 2022	S	DVS-321 Kenya 2021 (99.70%)	C
TF47 Kabara Uganda 2022	S	DVS-321 Kenya 2021 (99.70%)	C
194 Uganda 2022	S	DVS-321 Kenya 2021 (99.70%)	C
123 Uganda 2022	S	Sudan86 Sudan (99.17%)	C
NAK/1/31 Uganda 2022	S	DVS-321 Kenya 2021 (99.70%)	C
NAK/1/40 Uganda 2022	S	DVS-321 Kenya 2021 (99.70%)	C
	L	DVS-321 Kenya 2021 (99.75%)	C

^a^ NT: nucleotides.

**Table 5:** Amino acid differences among RVFV samples in cattle in Uganda.

Protein	Position	Samples					
		Lantana Uganda 2022	TF47 Kabara Uganda 2022	194 Uganda 2022	123 Uganda 2022	NAK/1/31 Uganda 2022	NAK/1/40 Uganda 2022
NSs	33	F	F	F	Y	F	F
	133	S	S	S	N	S	S
	152	A	A	A	T	A	A
	217	A	A	A	T	A	A

## Data Availability

Data is available upon reasonable request from the first author.

## Supplementary Materials

The following supporting information can be downloaded at: https://www.mdpi.com/article/10.3390/pathogens14070720/s1, Table S1: GPS Coordinates for RVF sample collection sites in Uganda.
