# Supplementary material for: Detection and Molecular Characterization of Rift Valley Fever Virus in Apparently Healthy Cattle in Uganda
Source: Pathogens. 2025 Jul 20;14(7):720. doi: 10.3390/pathogens14070720 (PMC12299453; doi:10.3390/pathogens14070720)
Supplement: Supplementary file 1 [file pathogens-14-00720-s001.zip › pathogens-3746545-supplementary.pdf]

pathogens

MDPI

Supplementary Materials

Table S1. GPS Coordinates for RVF sample collection sites in Uganda.

SN	DISTRICT	SUBCOUNTY	PARISH	VILLAGE	GPS
1	Gomba	Kyayi	Kyayi	Kibona	N: 00.20386; E: 031.43167
***2**	**Gomba**	**Maddu TC**	**Maddu**	**Maddu A**	**N: 00.19523; E: 031.43440**
3	Gomba	Maddu TC	Maddu	Namabaale	N: 00.22263; E: 031.67833
4	Gomba	Kabulasoke	Kisozi	Kijojo	N: 00.14997; E: 031.67879
5	Gomba	Maddu	Maddu TC	Kawala	N: 00.1500; E: 031.67881
6	Gomba	Maddu	Maddu TC	Kayunga	N: 002.23596; E: 031.72020
7	Gomba	Maddu	Kigezi	Kiwangula	N: 00.26714; E: 031.61488
8	Gomba	Maddu	Kawala	Kawala	N: 00.22891; E: 031.64963
9	Gomba	Maddu	Kigezi	Kikonoka	N: 00.27140; E: 031.62977
10	Gomba	Kabulasoke	Bulwada	Mataka	N: 00.19200; E: 031.75866
11	Gomba	Kabulasoke	Bulwada	Kiziko	N: 00.19140; E: 031.76613
12	Gomba	Kabulasoke	Bulwada	Kabankoni	N: 00.18272; E: 031.74648
13	Gomba	Kabulasoke	Bulwada	Kabankoni	N: 00.18660; E: 031.73263
14	Gomba	Kabulasoke	Bulwada	Kabankoni	N: 00.18243; E: 031.74767
15	Gomba	Kabulasoke	Bulwada	Kalungu	N: 00.18888; E: 031.75602
16	Gomba	Kabulasoke	Bulwada	Kabankoni	N: 00.21495; E: 031.66840
17	Gomba	Maddu	Kigezi	Kawungezi	N: 00.25746; E: 031. 61482
18	Gomba	Maddu	Kigezi	Kikonoka	N: 00.27584; E: 031.62157
19	Gomba	Maddu	Maddu	Maddu	N: 00.22025; E: 031.67907
20	Gomba	Maddu	Ddegeya	Ddegeya	Lat:0.19767; Long:031:60532
21	Gomba	Maddu	Ddegeya	Ddegeya	Lat:0.17750; Long:031:38420
22	Gomba	Maddu	Ddegeya	Kirasi	Lat:0.13912; Long:031.59528
23	Gomba	Maddu	Ddegeya	Kirasi	Lat:0.14442; Long:031.628462
24	Gomba	Maddu	Maddu	Maddu A	Lat:0.2222; Long:031.6447
25	Gomba	Maddu TC	Maddu Ward B	Kyamuyiisa	Lat: 0.18527; Long: 031.67494
26	Gomba	Maddu TC	Maddu Ward B	Maddu B	Lat: 0.20292; Long: 031.67155
27	Gomba	Maddu TC	Maddu Ward C	Kasambya Luyege	Lat: 0,25965; Long: 031.65415
28	Gomba	Maddu TC	Maddu Ward C	Kinogozi	Lat: 0.23516; Long: 031.71342
29	Gomba	Kabulasoke	Kisozi	Kisozi	N 00.06908; E 031.41163
30	Gomba	Kabulasoke	Kifampa	Katonga	Lat: 0.100949; Long: 031.638167
31	Gomba	Kabulasoke	Bulwadda	Bulwadda	N 00.11392; E 031.47144
32	Gomba	Maddu TC	Maddu Ward B	Kyamuyiisa	Lat: 0.18333; Long: 031.66667
33	Gomba	Maddu	Maddu	Maddu A	Lat: 0.17750; Long: 31.3842
34	Gomba	Kyayi	Kyayi	Kibona	N: 2.87583; E: 032.27893
35	Isingiro	Isingiro TC	Masha	Kyamisokya	S: 00.75071; E: 030.77636
36	Isingiro	Isingiro TC	Kaharo	Masha	S: 00.80488; E: 030.80555
37	Isingiro	Isingiro TC	Mabona	Kigarama	S: 00.85660; E: 030.77609
38	Isingiro	Isingiro TC	Kyabishaho	Rwenkuba	S: 00.80800; E: 030.79815
39	Isingiro	Isingiro TC	Mabona	Kyabirukwa	S: 00.80799; E: 030.78916
40	Isingiro	Isingiro TC	Mabona	Kyabirukwa	S: 00.85628; N: 030.77587
41	Isingiro	Masha	Nyakakoni	Rwembogo	S: 00.72795; N: 030.74191
42	Isingiro	Masha	Nyakakoni	Rwembogo	S: 00.43163; N: 030.45318
43	Isingiro	Masha	Nyakakoni	Rwembogo	S: 00.72803; N: 030.74215
44	Isingiro	Masha	Nyamitsindo	Buyojwa	S: 00.42898; N: 030.45960
45	Isingiro	Masha	Nyakakoni	Rwembogo	S: 00.71107; N: 030.76844
46	Isingiro	Masha	Nyakakoni	Rwembogo	S: 00.72820; N: 030.76027
47	Isingiro	Isingiro TC	Kaharo	Masha	S:00.74769; N: 030.76629
48	Isingiro	Isingiro TC	Kaharo	Masha	S: 00.72820; N: 030.76020
49	Isingiro	Masha	Nyamisindo	Buyojwa II	N: 03.01809, E: 030.91268
50	Isingiro	Masha	Nyamisindo	Buyojwa	S: 00.72164; E: 030.78201
51	Isingiro	Kabingo	Kabingo	Byaruka	S: 00.76790; E: 030.74115

52	Isingiro	Kabingo	Kyeirumba	Nyabugando	S: 00.75745; E: 030.75622
53	Isingiro	Kabingo	Kyeirumba	Nyabugando	S: 00.75511; E: 030.75972
54	Isingiro	Kabingo	Kyeirumba	Nyabugando	S: 00.75008; E: 030.75790
55	Isingiro	Kabingo	Kyarugaju	Rwemigango	S: 00.75726; E: 030.76541
56	Isingiro	Kabingo	Kyarugaju	Keminya	S: 00.70629; E: 030.83072
57	Isingiro	Kabingo	Kyarugaju	Keminya	S: 00.70193; E: 030.84595
58	Isingiro	Kabingo	Kagogo	Nyakagyera	S: 00.70252; E: 030.84514
59	Isingiro	Isingiro TC	Isingiro TC	Isingiro TC	S: 00.79573; E: 030.82260
60	Isingiro	Isingiro TC	Kamuli	Ruhimbo	S: 00.71573; E: 030.73262
61	Isingiro	Isingiro TC	Kamuli	Rwengiri	S: 00.79482; E: 030.81821
62	Isingiro	Isingiro TC	Kamuli	Rwengiri	S: 00.79389; E: 030.82280
63	Isingiro	Masha	Nyakakoni	Masha Cell	S: 00.71575; E: 030.73265
64	Isingiro	Masha	Nyakakoni	Nyakakoni B	S: 00.69213; E: 030.73040
65	Isingiro	Masha	Nyakakoni	Nyakakoni A	S: 00.69102; E: 030.73022
66	Isingiro	Kabingo	Kyeirumba	Kabagabe	S: OO.77127; E: 030.74525
67	Isingiro	Kabingo	Kyeirumba	Nyabugando	S: 00.77136; E: 030.74526
68	Isingiro	Kabingo	Kyeirumba	Nyabugando	S: 00.7154; E: 030.76020
69	Kiruhura	Nyakashashara	Rurambira	Nyakashashara	Lat: 0.52082; Long:31.06658
70	Kiruhura	Nyakashashara	Nyakahita	Nyakahita	Lat:0.48951; Long:31.050565
71	Kiruhura	Nyakashashara	Rurambira	Kakyera	Lat: 0.52081; Long:31:06544
72	Kirihura	Akayanja	Rwakobo	Rwekishwaga	Lat:0.52723; Long:31.00634
73	Kirihura	Akayanja	Rwakobo	Rwekishwaga	Lat:0.52787; Long:31.0031
74	Kirihura	Akayanja	Bushoro	Bushoro	Lat: 0.51775; Long:30.93866
75	Kirihura	Akayanja	Bushoro	Biira	Lat: 0.50018; Long:30.95646
76	Kirihura	Kanyaryeru	Rwamuranda	Rwamuranda	Lat:0.55697; Long:30.86734
77	Kirihura	Kanyaryeru	Rwamuranda	Obwapa Cell	Lat: 0.4992; Long:30.90461
78	Kirihura	Kanyaryeru	Rwamuranda	Obwapa	Lat:0.55622; Long:30.86092
79	Kirihura	Kanyaryeru	Kibega	Mpanga Mushanju	Lat:0.52597; Long:30.84923
80	Kirihura	Kanyaryeru	Kanyaryeru	Mpanga Mushanju	Lat:0.54083; Long:30.84194
81	Kiruhura	Sanga	Nombe	Nyakagando A	Lat:0.55103; Long:30.91028
82	Kiruhura	Sanga	Nombe	Rufuka	Lat:0.53546; Long:30.90077
83	Kiruhura	Nyakashashara	Rurambira	Rurambira	S:00.79647; E:030.82129
84	Kiruhura	Sanga TC	Nombe	Rufuka	S:00.52210; E:030.89912
85	Kiruhura	Sanga TC	Nombe	Rufuka	S:00.51885; E:030.89937
86	Kiruhura	Kanyaryeru	Kibega	Kibega	S:00.40562; E:030.86281
87	Kiruhura	Sanga	Nombe II	Nyankumba	S00.256851; E:030.58038
88	Kiruhura	Sanga	Akayanja	Rwakobo	S:00.30497;E:030.59728
89	Kiruhura	Sanga TC	Nombe Ward	Rufuka	S00.51881; E:03089936
90	Kiruhura	Nyakashashara	Rurambira	Rurambira	S:035.26; E:31.249
91	Kiruhura	Nyakashashara	Rurambira	Rurambira	S:030.327; E31.2437
92	Kiruhura	Nyakashashara	Rurambira	Rurambira	Lat:-0.5779; Long:31.0971
93	Kiruhura	Kanyaryeru	Rwamuranda	Rwamuranda	S:0.330; E:30.5257
94	Kiruhura	Kanyaryeru	Rwamuranda	Rwamuranda	S:0.3222; E:30.5241
95	Kiruhura	Kanyaryeru	Rwamuranda	Rwamuranda	S:0.320; E:30.5222
96	Kiruhura	Nyakashashara	Rurambira	Rurambira	Lat:0.5745; Long:31.05996
97	Kiruhura	Nyakashashara	Rurambira	Nyangakakoma	Lat:0.5883; Long:31.04757
98	Kiruhura	Nyakashashara	Nyakahita	Katengo	Lat:0.48466; Long:31.04793
99	Kiruhura	Nyakashashara	Nyakahita	Nyakahita	Lat:0.43646; Long:31.06708
100	Kirihura	Akayanja	Rwakobo	Rwekishwaga	Lat:0.53077; Long:31.00115
101	Kiruhura	Sanga TC	Nkongelo	Akabare	Lat:0.48977; Long:30.90784
102	Kiruhura	Sanga	Kakageti	Kasharara	Lat:0.53255; Long:30.91485
***103**	**Kiruhura**	**Kinoni**	**Naama**	**Naama**	**Lat:-0.16667, Long:31.01667**
***104**	**Kiruhura**	**Sanga**	**Sanga**	**Sanga**	**Lat:-0.48946, Long:31.04972**
105	Nakasongola	Wabinyonyi	Kageri	Kabugenda	N00.32153; E031.64167
106	Nakasongola	Wabinyonyi	Kageri	Kalobokwe	N01.32335; E032.50934
***107**	**Nakasongola**	**Wabinyonyi**	**Kikangula**	**Kikangula**	**N01.37727; E032.241191**
108	Nakasongola	Kakoge	Kyambogo	Wanjuki	N01.02834; E032.52899
109	Nakasongola	Wabinyonyi	Sikye	Lugogo	N01.28537; E032.35526
110	Nakasongola	Wabinyonyi	Sikye	Lugogo	N01.29986; E032.37857

111	Nakasongola	Kakoge	Kacunyire	Kalyakoti	N01.29990; E032.37856
112	Nakasongola	Wabinyonyi	Wampiti	Kalungu	N1.1339.2188; E32.2742.3432
113	Nakasongola	Wabinyonyi	Wampiti	Kalungu	N1.1821.1392; E32.3620.394
114	Nakasongola	Wabinyonyi	Wampiti	Kalungu	N1.1354.14772; E32.743.21872
115	Nakasongola	Migeera TC	Migerra West ward	Moslem zone	N01.40949; E032.24310
116	Nakasongola	Wabinyonyi	Wampiti	Kizongo	S00.48566; 032.86294
117	Nakasongola	Wabinyonyi	Wampiti	Kizongo	S00.30419; E032.55265
118	Nakasongola	Wabinyonyi	Kageri	Molwe	N01.36281; E032.47910
119	Nakasongola	Wabinyonyi	Kageri	Molwe	N01.36519; E032.47782
120	Nakasongola	Kakoge	Kakoge	Kibira Zone	N01.408033; E032.26383
121	Nakasongola	Migera TC	Migera TC	Pastor Zone	N01.40864; E032.25809
122	Nakasongola	Migera TC	Migera West Ward	Bizibitukura	N01.40868; E032.25811
123	Nakasongola	Migera TC	Migera West Ward	Moslem Zone	N01.40949; E032.24310
124	Nakasongola	Migera TC	Migera North Ward	Kyabacwezi	N01.36524; E032.47780
125	Nakasongola	Migera TC	Migera North Ward	Kyamukinda	N01.42050; E030.24504
126	Nakasongola	Kakoge	Kyeyindula	Kyeyindula	N01.03486; E032.30920
127	Nakasongola	Kakoge	Kyeyindula	Bukabi	N01.03901; E032.30767
128	Nakasongola	Kakoge	Kyeyindula	Nsolosolo	N01.05917; E032.30558
129	Nakasongola	Kakoge	Kyambogo	Kamuwandula	N00.58574; E032.33755
130	Nakasongola	Kakoge	Kyambogo	Kamuwandula	N00.58038; E032.32226
131	Nakasongola	Wabinyonyi	Kikangula	Kikangula	N01.36791; E032.44055
132	Nakasongola	Wabinyonyi	Sikye	Lugogo	N01.28539; E032.35529

Note: 1. Entries highlighted in red and marked with an asterisk (*) indicate sites from which RVF-positive samples were collected and subsequently sequenced. 2. TC = Town Council.
